# Optimizing multi-spectral ore sorting incorporating wavelength selection utilizing neighborhood component analysis for effective arsenic mineral detection

**DOI:** 10.1038/s41598-024-62166-0

**Published:** 2024-05-21

**Authors:** Natsuo Okada, Hiromasa Nozaki, Shinichiro Nakamura, Elsa Pansilvania Andre Manjate, Angesom Gebretsadik, Yoko Ohtomo, Takahiko Arima, Youhei Kawamura

**Affiliations:** 1 Division of Sustainable Resources Engineering, Graduate School of Engineering, Hokkaido University, Kita-13, Nishi-8, Sapporo, 060-8628 Japan; 2 Division of Engineering, Instituto Superior Politécnico de Tete, Tete, Mozambique; 3 Division of Sustainable Resources Engineering, Faculty of Engineering, Hokkaido University, Kita-13, Nishi-8, Sapporo, 060-8628 Japan; 4 Department of Mining Engineering, Aksum University, 7080 Aksum, Tigray Ethiopia

**Keywords:** Sensor-based ore sorting, Mineral processing, Neighborhood component analysis, Machine learning, System, Wavelength selection, Environmental sciences, Imaging and sensing, Optical spectroscopy, Scientific data

## Abstract

Arsenic contamination not only complicates mineral processing but also poses environmental and health risks. To address these challenges, this research investigates the feasibility of utilizing Hyperspectral imaging combined with machine learning techniques for the identification of arsenic-containing minerals in copper ore samples, with a focus on practical application in sorting and processing operations. Through experimentation with various copper sulfide ores, Neighborhood Component Analysis (NCA) was employed to select essential wavelength bands from Hyperspectral data, subsequently used as inputs for machine learning algorithms to identify arsenic concentrations. Results demonstrate that by selecting a subset of informative bands using NCA, accurate mineral identification can be achieved with a significantly reduced the size of dataset, enabling efficient processing and analysis. Comparison with other wavelength selection methods highlights the superiority of NCA in optimizing classification accuracy. Specifically, the identification accuracy showed 91.9% or more when utilizing 8 or more bands selected by NCA and was comparable to hyperspectral data analysis with 204 bands. The findings suggest potential for cost-effective implementation of multispectral cameras in mineral processing operations. Future research directions include refining machine learning algorithms, exploring broader applications across diverse ore types, and integrating hyperspectral imaging with emerging sensor technologies for enhanced mineral processing capabilities.

## Introduction

Mineral-intensive technologies such as renewable energy and electric vehicles will be in high demand as climate change is addressed and a sustainable energy future is transitioned. Despite this, the mining sector, especially in copper, is facing considerable difficulties due to the growing demand^1,2^. The depletion of high-grade ore and the rise of high-arsenic copper resources are more prominent issues in this field. Arsenic in low-grade copper ores not only makes mineral processing more difficult, but also causes environmental and health concerns due to the presence of arsenic in wastewater and exhaust gas^3,4^. The correlation between arsenic exposure and a range of health problems highlights the pressing requirement for ecologically viable methods in the mining sector. The incorporation of modern technologies such as hyperspectral imaging into sensor-based ore sorting systems has significant potential in this situation^5^. Ore sorting systems can successfully separate high-arsenic ores from valuable material by utilizing the precise and accurate analysis of mineral composition provided by hyperspectral imaging. This not only mitigates environmental dangers but also reduces processing costs. This strategy not only tackles the difficulties presented by ores containing high levels of arsenic, but also aids in the advancement of a mining industry that is environmentally friendly, in line with the objectives of the Paris Agreement.

Sensor-based ore sorting has become a crucial technology in mineral processing, providing numerous advantages that transform conventional mining methods. With sensor-based ore sorting systems, valuable minerals can be selectively extracted from ore streams according to their unique physical and chemical properties based on advanced sensor technologies. This process of selective extraction maximizes the efficient use of resources by effectively separating valuable ore from nonvaluable materials (or gangue minerals). In the field of mineral processing, Sensor-based ore sorting is a vital component as it enhances ore grades and minimizes the amount of waste material that is processed^6^. Evidence demonstrates that it effectively decreases the usage of energy, water, and reagents, while also minimizing the formation of fine waste, by disposing of trash before undergoing additional processing^7,8^. To successfully apply sensor-based sorting, it is crucial to select a sensing approach that can efficiently distinguish between ore and waste^9^. Sensor fusion, the integration of data from several sensing systems, has the potential to enhance the characterization of the detected material and improve sorting capability^4^. Microwave imaging (MWI) is a promising technique that can penetrate deeply into rock particles and serve as an additional approach for analyzing ores with significant differences in electromagnetic characteristics^10^. The efficacy of MWI in ore sorting has been validated by simulations and tests, affirming its capability to segregate valuable minerals or metals from unproductive particles. The utilization of sensor-based ore sorting presents substantial advantages in terms of reducing costs and enhancing efficiency in mineral processing.

### Hyperspectral imaging

Ore sorting techniques can be significantly enhanced by leveraging hyperspectral imaging technology, which offers unparalleled capabilities for mineral characterization and classification. Hyperspectral imaging allows ore sorting systems to analyze the distinct spectral fingerprints of minerals over a wide range of wavelengths, unlike traditional sorting methods that only consider physical attributes like size, shape, and density. This enables the identification and differentiation of minerals by analyzing their unique chemical compositions and optical features. Hyperspectral imaging is used in sensor-based ore sorting to analyze ore streams in real-time without causing damage^5^. This technique offers important details on the mineralogy and quality of the material being processed. By using hyperspectral imaging technology into sorting systems, mining companies can enhance their efficiency, precision, and selectivity in segregating valuable minerals from waste material. As a result, mineral processing enterprises have higher rates of recovery, lower costs of processing, and increased profitability.

### Curse of dimensionality and feature reduction

The processing of hyperspectral data is more challenging than that of other types of data due to the sheer volume of information collected, which may be affected by issues with its dimensions. High-dimensional spectral bands in hyperspectral images are often highly similar, which makes them susceptible to the "curse of dimensionality," a phenomenon that affects many traditional algorithms^11^. Within the domain of hyperspectral ore sorting systems, the notion of wavelength selection arises as a crucial strategy for enhancing sorting efficiency and precision. Wavelength selection is the process of strategically identifying and using wavelengths of electromagnetic radiation (light) that provide the most useful information for differentiating between various minerals or compounds in an ore stream. Through the analysis of distinct spectral patterns displayed by minerals at different wavelengths, the process of wavelength selection allows ore sorting systems to concentrate on the specific spectral bands that are most effective in distinguishing the desired minerals. By employing this focused method, the precision, effectiveness, and dependability of mineral identification and separation procedures are enhanced, resulting in better utilization of resources and increased operational performance in mineral processing. The process of choosing the right wavelength is also extremely important to reduce the likelihood of incorrect positive and negative results, maximize the rate at which valuable minerals are recovered, and to reduce the waste stream losses of potentially valuable materials. The significance of ore sorting lies in its ability to facilitate efficient and precise separation of valuable ore from waste or gangue materials. Based on their unique reflectance or absorption properties, sensors can effectively distinguish ore from gangue by using specific wavelengths, such as visible or mid-infrared ones. This enhances the system's ability to choose and efficiently sort materials, especially when working with intricate ores or comparable substances. Utilizing wavelength selection can improve the ability of photometric sensors to distinguish between different substances and simplify the creation of new sensors for the purpose of sorting ores and characterizing minerals^12^. A variety of advanced techniques are used to analyze multidimensional spectrum data and extract relevant features from hyperspectral data, such as spectral features extraction and machine learning algorithms i.e. linear regression, K-means clustering, neural network^13–15^.

The intricate nature and extensive dimensions of multi-spectral data require the application of sophisticated data classification techniques such as Neighborhood Component Analysis (NCA). Advanced data classification techniques like NCA are needed to handle multi-spectral data due to several reasons. To begin with, hyperspectral data typically includes a substantial number of spectral bands, which might provide computing difficulties during processing and analysis^16^. The issue can be addressed by using NCA, which involves lowering the dimensionality of the data. This would lead to improved processing and classification efficiency^17^. Additionally, it is essential to note that conventional classification methods designed for multispectral data may not be appropriate for hyperspectral data, as the latter offers more intricate and comprehensive spectral information^18^. The NCA method can effectively handle hyperspectral data with a high number of dimensions. It achieves improved classification accuracies by taking into account both spectral and spatial information^19^. Additionally, NCA offers advantages such as low computational requirements and shorter classification times^20^. Therefore, advanced techniques like NCA are essential for accurately classifying hyperspectral data while overcoming the challenges associated with high dimensionality and detailed spectral information.

In this study, Neighborhood Component Analysis (NCA) was applied as a preprocessing step to reduce the dimension of Hyperspectral (HS) data of arsenic-bearing minerals by identifying several wavelength bands important for mineral identification. Then the identified wavelength bands were used as inputs to train machine learning algorithms for identifying Arsenic (As) minerals concentration in simulated ore materials. Multispectral (MS) cameras are more cost-effective and provide faster data collecting and processing compared to HS cameras; hence, they are projected to enable mineral identification utilizing data from a few wavelength bands. The HS data of arsenic-bearing minerals (enargite) were used NCA a machine learning method, as a band selector, and identified several wavelength bands important for mineral identification. Then, the data containing only the minimum number of wavelengths were analyzed for identification of mineral contents/ratios using machine learning algorithms. These will improve the selectivity of wavelengths, considering the ore characteristics produced by each mine. In addition, the application of the herein proposed machine learning algorithm for HS images analysis is expected to improve the efficiency of ore selectivity, i.e. improve the speed of the ore sorting process.

### Literature review

To develop environmentally sustainable resources, it’s essential to develop advanced metal recovery technology for these high-grade arsenic ores, and Sensor-Based Ore Sorting (SBOS) can achieve this. SBOS, when implemented as a presorting process before the normal beneficiation process, can reduce the amount of ore that must be processed to produce a certain amount of value-added metal, which has a significant impact on the economics of the mine and the plant as a whole^21–23^. It can also reduce the environmental impact by reducing the tailings produced in the subsequent beneficiation process. Non-destructively classified tailings are geotechnically stable and can be easily stored due to their low moisture content^24^. Robben and Wotruba highlighted that the introduction of SBOS would have an impact on both the environmental and economic aspects of the mineral processing process^25^. However, the authors pointed out that SBOS is still in the market entry stage of the mineral industry and further technological development is required.

Mineral analysis requires knowledge of crystallography as well as chemical analysis^26^. However, the methods commonly used for mineral analysis, such as Electron Probe Micro Analyzer (EPMA), X-ray diffraction (XRD) and Scanning Electron Microscope (SEM), are relatively time-consuming and depend on experience^27^, they are not realistic in terms of identification speed, convenience, and economy when used in actual mineral processing operations.

Therefore, SBOS has been developed as a form of mineral identification suitable for beneficiation. In recent years, more and more equipment has been installed that can withstand larger production scales^25^. SBOS methods have utilized a range of sensing technologies, including X-ray transmission, X-ray fluorescence, optical sensing, and inductive sensing^9,10,28^. Furthermore, the utilization of area-scan cameras and predictive tracking systems that rely on machine learning approaches have demonstrated potential in minimizing characterization and separation errors^29^. Researchers have also studied the combination of data from several sensing approaches to improve the sorting ability of these systems^6^. While different SBOS methods have been developed and introduced particularly focusing on SWIR data, there are few studies or methods on mineral identification/sorting using VNIR short-wavelength HS data. However, there is growing interest in visible to near-infrared (VNIR) spectroscopy for mineral identification, and in some recent studies VNIR wavelengths have been used to classify rocks and minerals^30,31^.

Sensor-based ore sorting methods and technologies have the potential to significantly improve ore grades and reduce waste in mineral processing^6^. These methods, which rely on the electromagnetic spectrum, can be classified based on their characteristics and limitations^28^. An example of a successful method for sorting complicated ores is the utilization of hyperspectral short-wave infrared sensors in conjunction with machine learning, as demonstrated by Tusa^32^. Sensor-based ore sorting can be applied at various stages in the process flow diagram, making it a versatile and valuable tool in the mining industry^33^.

In the field of remote sensing, mineral identification in the near-infrared region has been widely used^34,35^ and they have shown excellent performance in ore classification. On the other hand, the high cost of HS cameras and the time required for data acquisition have been barriers to their application in actual operations, where immediate classification is required. In a previous study^24,36^, HS data of minerals were analyzed by deep learning to identify minerals. The use of deep learning allows the creation of more versatile and simplified learning models compared to conventional machine learning or identification methods that combine multiple machine learning models. However, since HS images consist of several hundred spectral bands, there is a high correlation between proximity spectra, and data analysis without preprocessing is highly redundant and computationally intensive. Therefore, dimensionality reduction is necessary as a preprocessing step for a large amount of data to be generated.

Dimensionality reduction methods for HS commonly fall into two categories: band extraction and wavelength selection. The band extraction methods map a high-dimensional feature space to a low-dimensional space; therefore, cannot preserve the original physical interpretation of the image and is not applicable as a dimensionality reduction method^37^. While the wavelength selection method can maintain the original physical interpretation of the images. According to a review by Sun and Du^38^, wavelength selection methods can be categorized into six groups: ranking-based methods, searching-based methods, clustering-based methods, sparsity-based methods, embedding learning-based methods, and hybrid scheme-based methods.

A variety of studies have explored different techniques for wavelength selection and spectral data classification in mineral processing. Ghosh^39^ introduced an infrared thermography-based method for sorting alumina-rich iron ores, while Kern^40^ suggested utilizing short-wavelength infrared and dual-energy X-ray transmission sensors for the Hammerlein Sn–In–Zn deposit. Phiri^41^ investigated the potential of near-infrared sensors for separating carbonate-rich gangue from copper-bearing particles in a copper–gold ore sample. Tusa^32^ advanced the field by evaluating hyperspectral short-wave infrared sensors, combined with machine learning methods, for pre-sorting complex ores. These studies collectively illustrate the potential of various wavelength selection techniques for enhancing the efficiency and effectiveness of ore sorting systems.

Furthermore, numerous research endeavors have investigated the implementation of machine learning algorithms to automate the task of wavelength selection and spectral data classification. Passos^42^ introduced an automated deep learning pipeline to optimize neural architecture and hyperparameters for spectral classification. Duan^43^ proposed a template matching approach achieving high accuracy without training, while Wang^44^ developed a multifunctional optical spectrum analysis technique utilizing support vector machines for optimal accuracy and speed. Baskir^45^ presented a MATLAB toolbox for user-friendly wavelength selection and automated spectral region selection. These investigations collectively underscore the potential of machine learning in automating and enhancing the process of wavelength selection and spectral data classification.

In addition to this, Advancements in hyperspectral imaging technology have significantly expanded the potential applications of this technology^46^. However, the complexity of hyperspectral data, including its high dimensionality and size, requires innovative methodologies for effective processing and analysis^47^. These challenges have led to the development of a range of image processing and machine learning analysis pipelines^46^. Notably, hyperspectral imaging finds application in microscopy, enabling the capture and identification of different spectral signatures in a single-pass evaluation^48^.

The effectiveness of machine learning algorithms, particularly Neighborhood Component Analysis (NCA), for multi-spectral data classification in mineral processing has been highlighted in recent research. Jahoda^49^ and Sinaice^50^ both emphasize the advantages of combining spectroscopic methods with machine learning for mineral identification. Jahoda^49^specifically highlights the superiority of machine learning methods in this context, while Sinaice^50^ proposes a system integrating hyperspectral imaging, NCA, and machine learning for rock and mineral classification. These findings are further supported by Carey^51^, who stresses the importance of spectrum preprocessing and a weighted-neighbors classifier for optimal mineral spectrum matching performance.

In their previous study Okada et al.^24^, developed a basic technology of SBOS, using hyperspectral (HS) imaging and deep learning as an expert system for mineral identification. HS is promising as SBOS to avoid As-containing copper minerals technic instead. In that study, HS imaging was used as a sensor to collect the intensity of wavelength data, which was then used to train deep learning algorithms for mineral identification. The HS image is cube-shaped data with dimensions in the wavelength and spatial directions, with wavelength data from the visible to near-infrared regions (400 ~ 1000 nm, 204 bands). Minerals (hematite, chalcopyrite, galena) identification was performed by analyzing detailed wavelength data of 204 bands in the shorter wavelength range of 400–1000 nm (from the visible light region to a part of the near-infrared region) using deep learning. However, the HS data used in that study consisted of 204 high-dimensional data, which required heavy computational resources. In addition, the HS camera itself is expensive, which was a barrier to its introduction/implementation in the operating site (mineral processing plant).

Yokoya and Iwasaki^52^ reported that, since each pixel provides continuous electromagnetic spectral characteristics, it’s possible to obtain detailed information about the target object. Owing to the high spectral resolution HS imaging is applied in fields such as remote sensing and quality control of food deep and pharmaceuticals. Robben et al.^53^ pointed out that, minerals show specific absorption characteristics in the near-infrared region from 1300 to 2550 nm due to vibrations of the bonding molecules contained in each mineral. A skilled expert can identify some minerals visually (Color), and the continuous electromagnetic spectrum in the short wavelength region is considered to contain optical data with mineral-specific physical properties.

Sorting machines that use MS images with a reduced number of dimensions are now technically feasible. They sort by acquiring specific wavelength information predetermined for each ore type. However, even for the same type of mineral, there are subtle differences in the formation and shape of each mine that affect the spectra. Additionally, the light environment inside each plant varies, which also affects the spectrum. Based on these factors, it is suggested that ore selectivity could be improved by defining the wavelength to be acquired for each ore type. To achieve this, we propose a framework that allows for the selection of spectral bands based on the characteristics of the ore produced. This framework will greatly support the tuning of the sorting process. As a case study, we will use a mineral sample containing arsenic.

The literature review highlights various gaps in current mineral processing practices, emphasizing the need for innovative approaches to improve efficiency and sustainability. While Sensor-Based Ore Sorting (SBOS) offers promise for environmentally friendly metal recovery, further technological development is required to enhance its effectiveness and practicality in operational settings. Traditional mineral analysis methods are time-consuming and impractical for real-time processing, prompting the exploration of faster and more economical techniques. Additionally, the application of machine learning algorithms and hyperspectral imaging for mineral identification presents computational challenges and limitations in the practical implementation due to the high dimensionality of data.

In response to these challenges, the proposed framework integrates Neighborhood Component Analysis (NCA) and machine learning algorithms to address the complexities of mineral identification and sorting using multi-spectral data. By reducing data dimensionality and identifying crucial wavelength bands, the framework enables efficient mineral identification while considering the unique characteristics of each ore type. Furthermore, by utilizing multi-spectral cameras with reduced dimensions, the framework enhances sorting efficiency and selectivity, paving the way for more sustainable mining practices and improved operational outcomes in mineral processing plants. In this study, a clustering-based method, Neighborhood Components Analysis (NCA), was used to perform dimensionality reduction and wavelength selection on HS data. After the selection, the selected bands were learned by machine learning algorithms to experiment with mineral identification. It is expected that mineral identification using fewer wavelengths than HS data will enable data acquisition with less expensive MS cameras and increase the efficiency of mineral identification.

## Methodology

**Figure 1:**
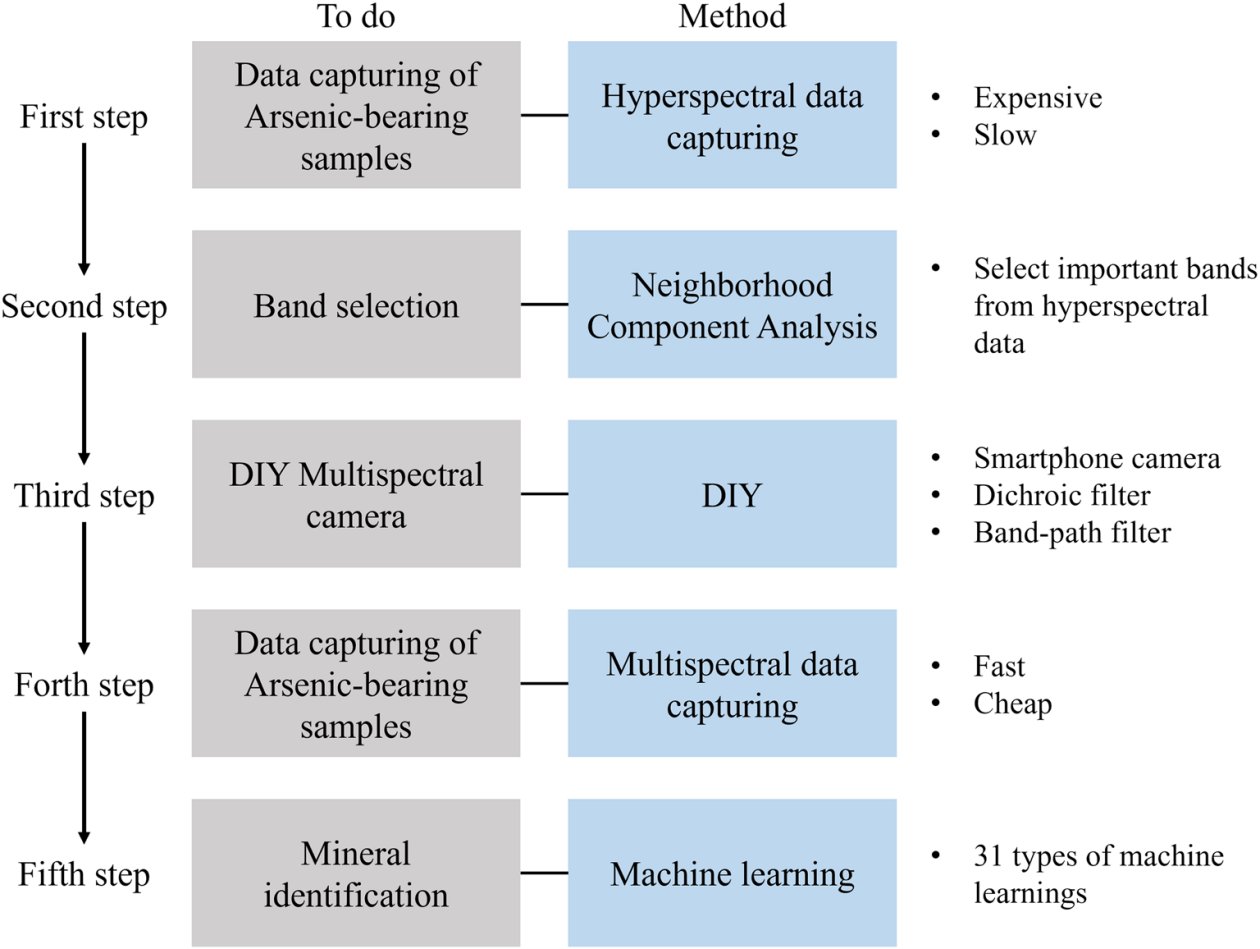
A summary of a flowchart of the research.

This study was conducted according to the flowchart in Fig. 1. After acquiring hyperspectral data, dimensionality reduction is performed using NCA to enable mineral identification using a multispectral camera.

### Methodology for hyperspectral (HS) imaging

A HS camera is a camera that can acquire intensity of wavelength data with more than 100 spectrally oriented wavelength bands in addition to two-dimensional planar data. Spectral Imaging's Specim IQ^54^, used in this study, can capture the intensity of wavelengths from 400 to 1000 nm. It is capable of spectroscopy in 204 wavelength bands and can capture continuous electromagnetic spectra for each wavelength of approximately 3 nm. Minerals are thought to have a unique intensity of wavelength patterns, and these characteristic intensities of wavelength patterns were learned using machine learning.

### Bands selection using neighborhood component analysis (NCA)

**Figure 2:**
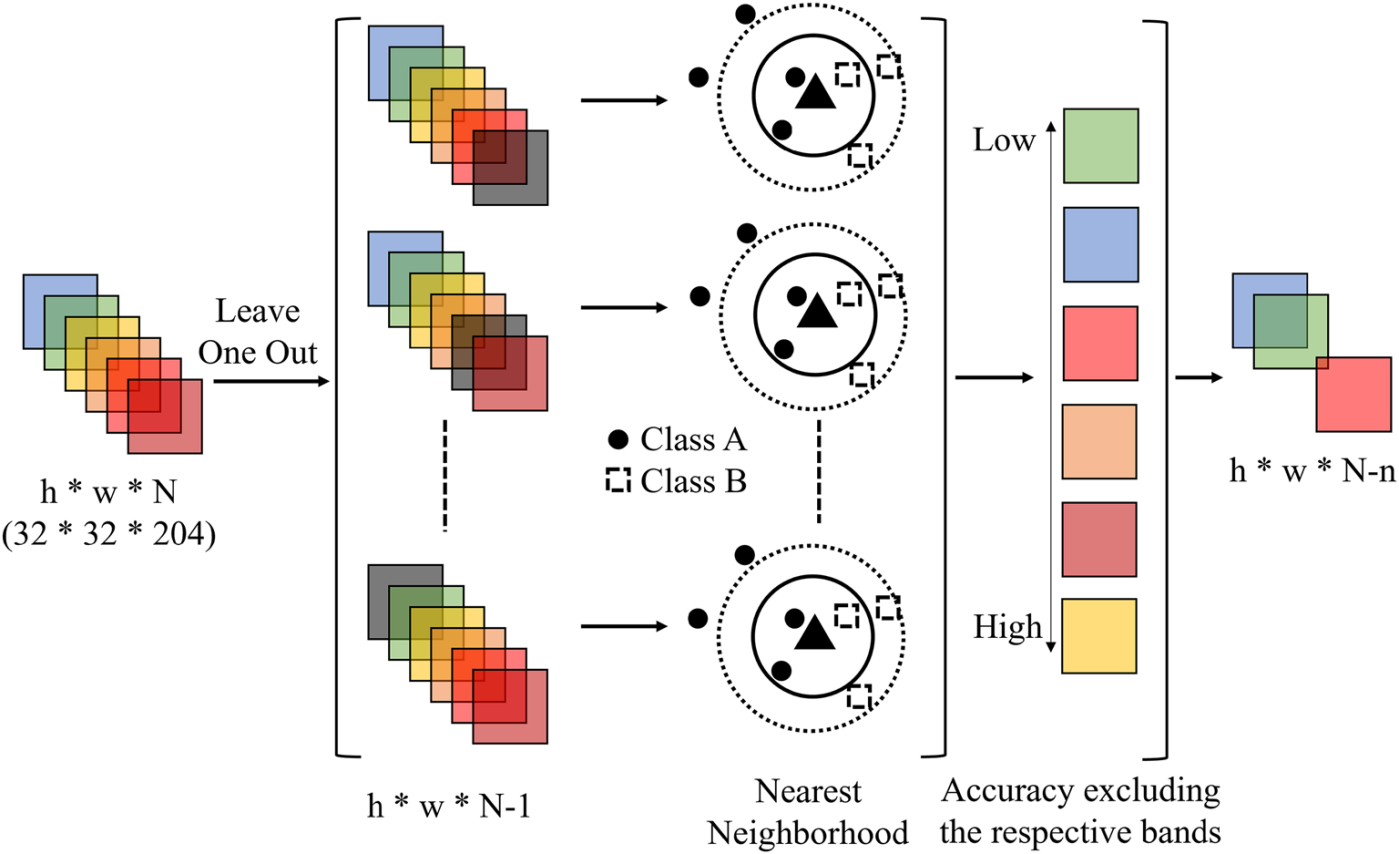
An overview of the NCA algorithm, which combines the Leave-One-Out and neighborhood methods to indicate the importance of the input bands.

The high redundancy of HS data and the resulting high dimensional data are fatal flaws in SBOS, which require high-speed ore sorting. Therefore, by using NCA, only the most important wavelength bands of HS data are selected, and the MS sensor installed in SBOS acquires only those bands for faster analysis. Figure 2 shows the flow of wavelength selection by NCA.

NCA is a type of machine learning algorithm/technique used for wavelength selection as well as clustering. It is a nonparametric process for a dataset and does not require parametric assumptions such as normal distribution. The data learning method uses a data classification method called Leave-One-Out (LOO), in which only one example is extracted from a set of *n* data as test data and the rest as training data, and the validation is repeated so that all the data become test data one at a time. Index number is represented as *i*. In NCA, the LOO probability was calculated to optimize the distance function with the weight as a function of probability *p*, as shown in Eq. (1) ^55,56^.

1 $$F\left(w\right)=\frac{1}{n}\sum_{i=1}^{n}{p}_{i}$$

Here, transforming the right-hand side of *F(w)* which is the function of weight *w*, is shown as in Eqs. (2)–(4).

2 $$\begin{array}{c}F\left(w\right)= \frac{1}{n}\sum_{i=1}^{n}{p}_{i}-\lambda \sum_{r=1}^{p}{w}_{r}^{2}\end{array}$$

3 $$\begin{array}{c}F\left(w\right)= \frac{1}{n}\sum_{i=1}^{n}\left[\sum_{j=1, j\ne i}^{n}{p}_{ij}{y}_{ij}-\lambda \sum_{r=1}^{p}{w}_{r}^{2}\right]\end{array}$$

4 $$F\left(w\right)=\frac{1}{n}\sum_{i=1}^{n}{F}_{i}\left(w\right)$$

To obtain the weight vector *w*, *f(w)* = *-F(w)* and *f*_*i*_*(w)* = *-F*_*i*_*(w)*, as in Eq. (5) below. The weight vector *w* was obtained by analyzing HS data with NCA to identify the wavelength bands that are important for mineral identification.

5 $$\widehat{w}=argminf\left(w\right)=argmin\frac{1}{n}\sum_{i=1}^{n}{f}_{i}\left(w\right)$$

In this study, the formulas shown in Eqs. (1) through (3) were run using MATLAB. NCA was performed on hyperspectral data. The weights were calculated by Eq. (3).

### Machine learning

Edgar and Manz^57^ defined machine learning as a field of study that looks at using computational algorithms to turn empirical data into usable models. In this study, the wavelength bands that are important for mineral identification among the mineral wavelength data were selected using NCA. These bands were then used as input to train a total of 33 different types of machine learning algorithms, as shown in Table 1. The use of a variety of machine learning algorithms allows for a comprehensive evaluation of overall performance. However, it is important to address the concerns raised regarding the number of classifiers and the parameter configurations.

**Table 1:** Machine learning methods used for MS data learning.

Fine tree	Linear SVM	Cosine KNN	Medium neural network
Medium tree	Quadratic SVM	Cubic KNN	Wide Neural Network
Coarse tree	Cubic SVM	Weighted KNN	Bilayered Neural Network
Linear discriminant	Fine Gaussian SVM	Boosted trees	Trilayered Neural Network
Quadratic discriminant	Medium Gaussian SVM	Bagged trees	SVM Kernel
Efficient logistic regression	Coarse Gaussian SVM	Subspace discriminant	Logistic Regression Kernel
Efficient linear SVM	Fine KNN	Subspace KNN	
Gaussian Naive Bayes	Medium KNN	RUSBoosted Trees	
Kernel Naive Bayes	Coarse KNN	Narrow Neural Network	

A diverse set of 33 machine learning algorithms was employed to comprehensively evaluate their performance. Although the use of numerous classifiers might seem excessive, it was intended to ensure a thorough exploration of different methodologies. However, it is acknowledged that optimizing parameters for each classifier is necessary to achieve maximum accuracy. Instead of providing detailed parameter configurations, the number of wavelengths (from 1 to 100 bands) was varied to determine the optimal number of bands for each individual method. Furthermore, rigorous methods, including cross-validation, were employed to fine-tune the parameters of each classifier, thereby ensuring the robustness of the results.

## Case study and experimental procedures

**Figure 3:**
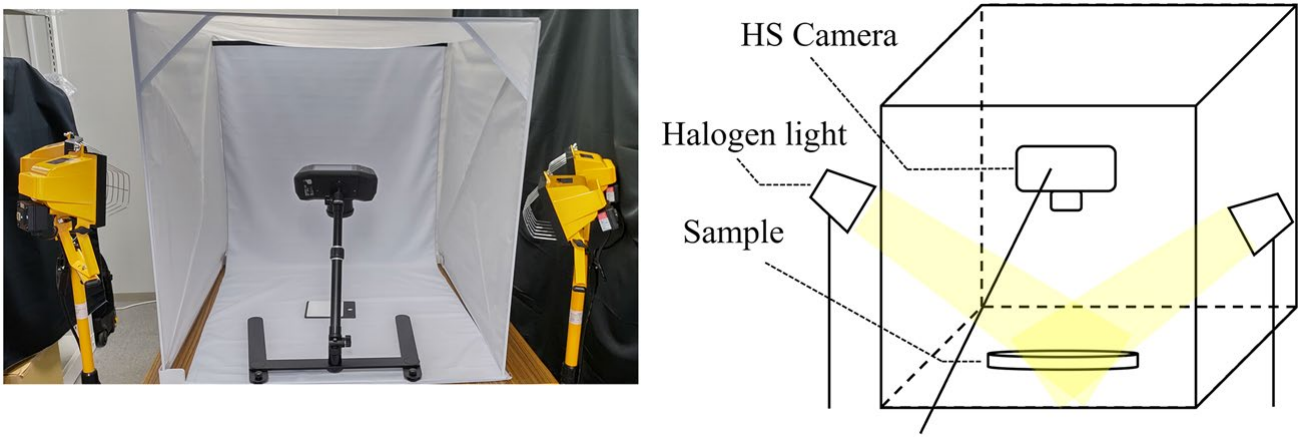
The sample is illuminated from two directions by a halogen light source, and the wavelength of the reflected light from the sample is acquired with a HS camera. The left figure shows the actual experimental scene, and the right figure shows the schematic diagram of the experiment.

Figure 3 shows the experimental situation and conceptual diagram of the imaging experiment. The experiments were conducted in a dark room to avoid light source interference. In addition, to avoid shadows on the sample, the target ore was illuminated from two directions by halogen lights from the left, and right of the camera. Halogen lights can uniformly illuminate the wavelength range from 500 to 3000 nm, which is the wavelength range used in this study. The filament of the halogen light heats up to a very high temperature as time passes, and the wavelength peak of the halogen light shifts to a higher wavelength. However, prior experiments confirmed that the effect was negligible.

**Figure 4:**
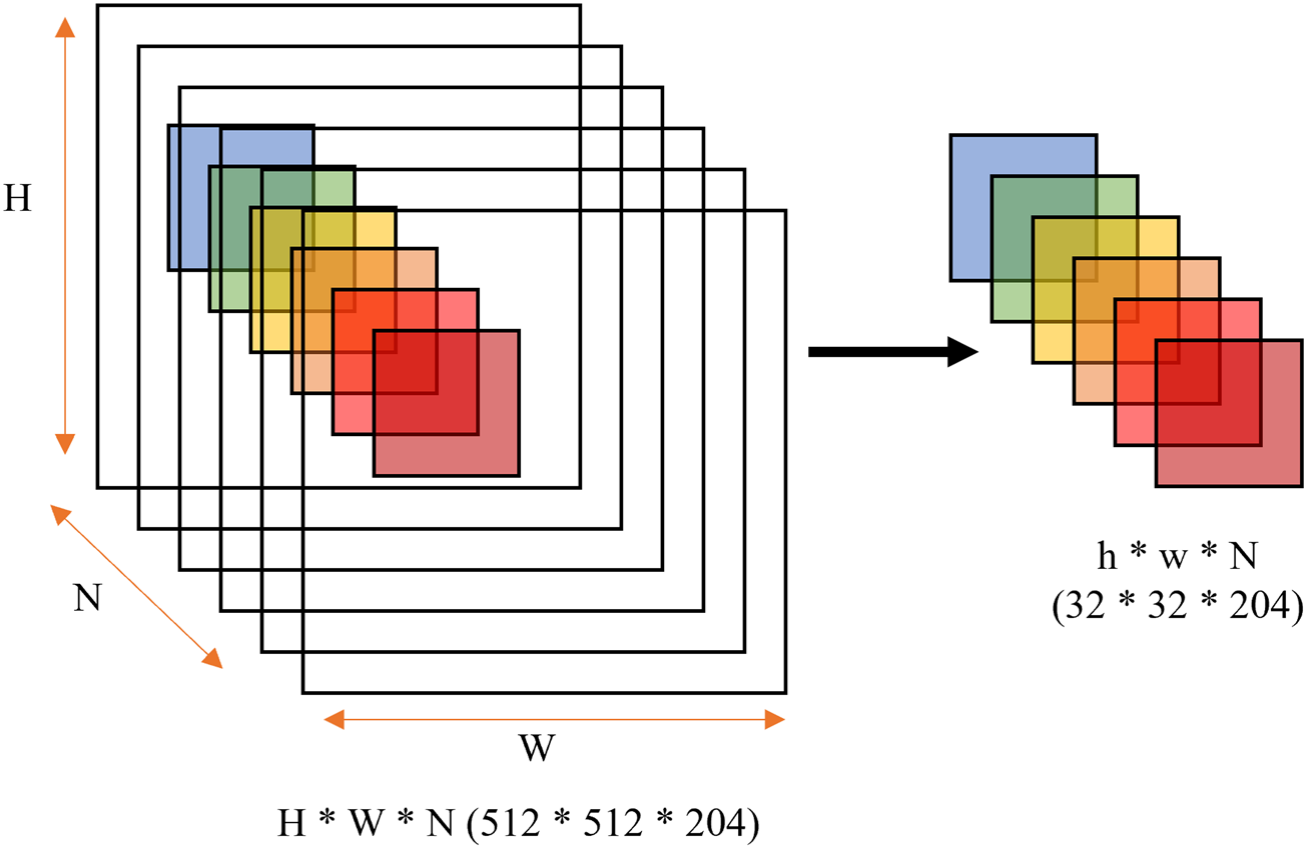
Pre-processing method for HS data, where H is height, W is width, and N is the number of bands. The data were cut out along the band direction to have height h and width w.

The surface of the powdered mineral was smoothed and photographed from 15 cm away. Although a single exposure takes about one minute, depending on the exposure time, the camera is heated strongly by the illumination;therefore, the camera was used for only about one hour. The illuminance of the light source was measured at each shooting with an illuminance meter. However, since the camera automatically adjusts the exposure time and corrects the illumination using a white plate, the effects of these factors were disregarded from the analysis test conducted beforehand. Before photographing the sample, it’s necessary to photograph a White Reference as a way of calibrating the camera. Thus, the intensity of wavelength data acquired in subsequent photography is obtained as the reflection intensity relative to the reflection intensity of the White Reference. The HS data taken by the camera can be shown as the intensity of wavelength data of wavelengths on the computer. The data acquired by a single HS camera is represented as a three-dimensional data cube with depth in the wavelength direction in addition to the vertical and horizontal quadratic planes. There are 512 pixels in the vertical plane, 512 pixels in the horizontal plane, and 204 pixels in the wavelength band that corresponds to the depth part. In this study, the vertical and horizontal pixels were divided into 16 equal parts to create 256 data cubes of 32 pixels each in the vertical and horizontal directions and 204 pixels in the depth direction. Each of these divided data cubes was then averaged to be 1 pixel in height and width, and the data set shown in Fig. 4 was prepared with 1 pixel in height and width and 204 pixels in depth.

### Target minerals for the experiment

**Figure 5:**
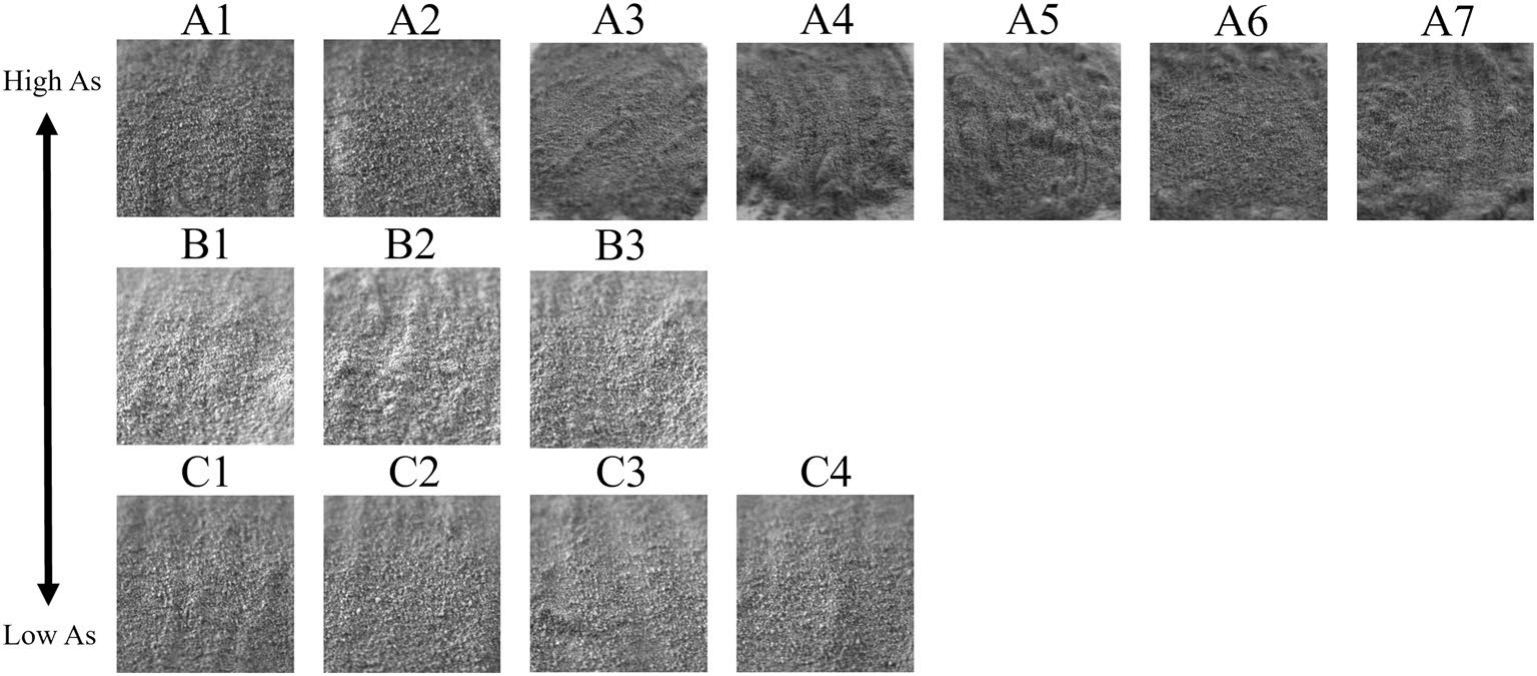
Samples images of 14 arsenic samples diluted by quartz.

Fourteen samples with different concentrations of arsenic, copper, and iron as shown in Table 2 were prepared by mixing powdered copper ore deposits collected from the Chuquicamata Mine^58^ and powdered quartz as shown in Fig. 5. The copper ore deposits comprise 76.98% of quartz and 8.74% of sulfide minerals (enargite, chalcopyrite, pyrite) with other minor components. The main host mineral for As is enargite Cu3AsS4. Ore samples with various grades were simulated by dilution of powdered quartz. By making the samples in powder form, it was possible to distribute the components evenly, and the acquired HS data were thought to contain the intensity of wavelengths of each mineral component evenly. Each sample was diluted with powdered quartz to achieve the same concentration (in weight ratio units) within the group. In smelters, a penalty occurs at arsenic concentrations of 0.2–0.5% or higher in the copper ore to be smelted/processed; therefore, it’s necessary to determine the arsenic concentration. The arsenic grades used as samples in this study were of the order of 0.2% to 0.5%, or arsenic concentration were aligned to investigate whether they are detectable for even lower As concentration. Group A was prepared for those with relatively high arsenic concentrations (avg. 0.23 wt%), Group B for those with moderate concentrations (avg. 0.10 wt%), and Group C for those with low arsenic concentrations (0.06 wt%). The chemical composition of each sample was determined using an Inductively Coupled Plasma (ICP) optical emission spectrometer (SPS-5510, Hitachi High-tech, Tokyo, Japan. SII became a 100% subsidiary of Hitachi High-tech in 2013). As, Cu, Fe and En (enargite) concentrations in each sample are shown in Table 2. After capturing HS data using the HS camera, the elements were used in machine learning.

**Table 2:** The chemical composition of each sample

Sample name	As [wt%]	Cu [wt%]	Fe [wt%]	En [wt%]
A1	0.30	5.27	2.29	1.58
A2	0.31	7.18	2.41	1.63
A3	0.27	6.01	1.45	1.41
A4	0.24	5.56	1.04	1.26
A5	0.26	5.40	1.08	1.38
A6	0.27	5.38	0.98	1.39
A7	0.24	4.91	1.00	1.27
B1	0.10	2.32	0.88	0.53
B2	0.12	1.75	0.80	0.64
B3	0.09	2.17	1.22	0.46
C1	0.07	0.90	0.65	0.36
C2	0.05	0.93	0.63	0.25
C3	0.08	0.81	0.60	0.41
C4	0.02	0.63	0.61	0.13

Group A contains high concentrations of arsenic, Group B medium concentrations, and Group C low concentrations.

### Acquired hyperspectral (HS) data

**Figure 6:**
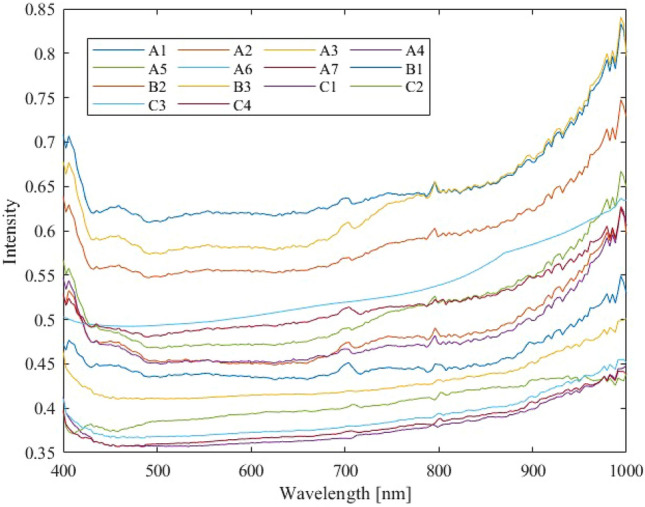
Distinctive protrusions appear at 700 nm and 800 nm; the spectral intensity tends to increase in the near-infrared region after 800 nm.

The averaged HS data of the test samples A, B, and C obtained by the HS camera (14 types in total) are shown in Fig. 6. The horizontal axis represents the wavelength (nm), the vertical axis represents the relative intensity normalized by White Reference, and each line represents the HS data obtained from the captured sample. Each HS line corresponds to the average of the data contained in 16 × 16 pixels, from A1 to C4, with different colors for each type. The overall trend is that the data is almost horizontal in the visible light region (400–800 nm), but characteristic protrusions are seen in some parts of the data. In the near-infrared region (800–1000 nm), the reflection intensity tends to increase. The reflection intensity at both ends of the spectrum is also higher. In other wavelength bands as well, the reflectance intensity shows many peaks of up and down fluctuations that cannot be observed by the human naked eye. In addition, the overall reflection intensity tends to increase as the quartz concentration increases, because the dark, low reflection intensity enargite was diluted with white quartz, which has a high reflection intensity. However, in the preliminary research^36^ using color images conducted beforehand, it was difficult to determine the color of the sample surface, i.e., only by the size of the reflectance intensity. In practical/field application of the SBOS, the light source environment varies depending on the location where the SBOS is installed, and the reflection intensity is thought to vary depending on the light source environment.

Although there are differences in the degree of peaking and the shape of the intensity of wavelengths depending on the spectral intensity of each sample, the basic shape of the wavelengths remains unchanged. These acquired data are now input to the machine learning algorithms which are trained to learn features of the data to identify As concentration.

## Result and discussion

### Analysis of machine learning using selected bands by neighborhood component analysis (NCA)

#### Bands selection using NCA

**Figure 7:**
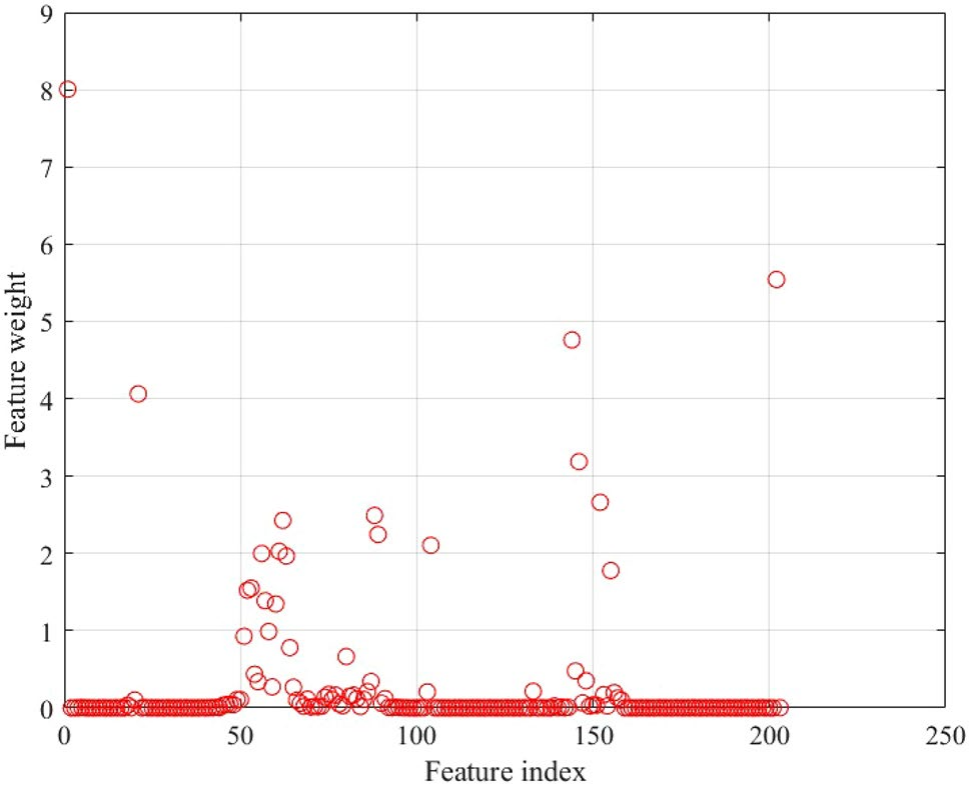
Shows each band weighted by NCA. The vertical axis shows feature weight and the horizontal axis shows the number of bands.

NCA was performed on the input HS data, and the weights, which indicate the importance of each wavelength band in mineral identification, were calculated as shown in Fig. 7. The vertical axis shows the feature weights, and the horizontal axis shows the wavelength band classifications from 1 to 204. A larger weight indicates that the data in that wavelength band is important, while a smaller weight indicates that the data is unimportant. In the figure, the feature weight is almost zero in the band bands 30 ~ 50 (488 ~ 547 nm), 120 ~ 130 (752 ~ 782 nm) 180 ~ 190 (929 ~ 958 nm). In other bands, the feature weight is more than 1, but it is not concentrated in some wavelength bands, but widely distributed in important wavelength bands. Considering the large amount of information and redundancy of HS data, the feature weight is low. Avoiding the analysis of wavelength data can save computational resources. Therefore, in this study, we analyze the wavelength data set with high feature weight (MS data) selected by NCA to perform data processing faster than analyzing HS data.

#### Multispectral (MS) data analysis

Using the selected intensity of wavelength data, mineral identification was performed with various machine learning algorithms as shown in Table 1. Since different combinations of machine learning algorithms and data sets used have different compatibility, in this study multiple machine learning algorithms were trained on the same data set to find the best performing combination or machine learning algorithm. From the wavelength selection by NCA, we created datasets with the top 25, 20, 15, 10, 9, 8, 7, 6, 5, 4, 3, 2, and 1 band each with high feature weight values. Then, each dataset was trained once with 33 different machine learning algorithms using the machine learning application MATLAB.

**Figure 8:**
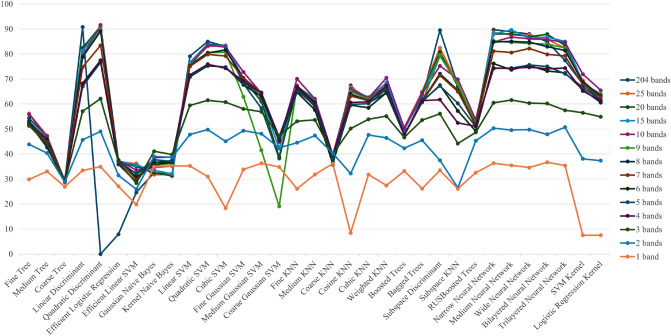
Machine learning results (Validation accuracy).

**Figure 9:**
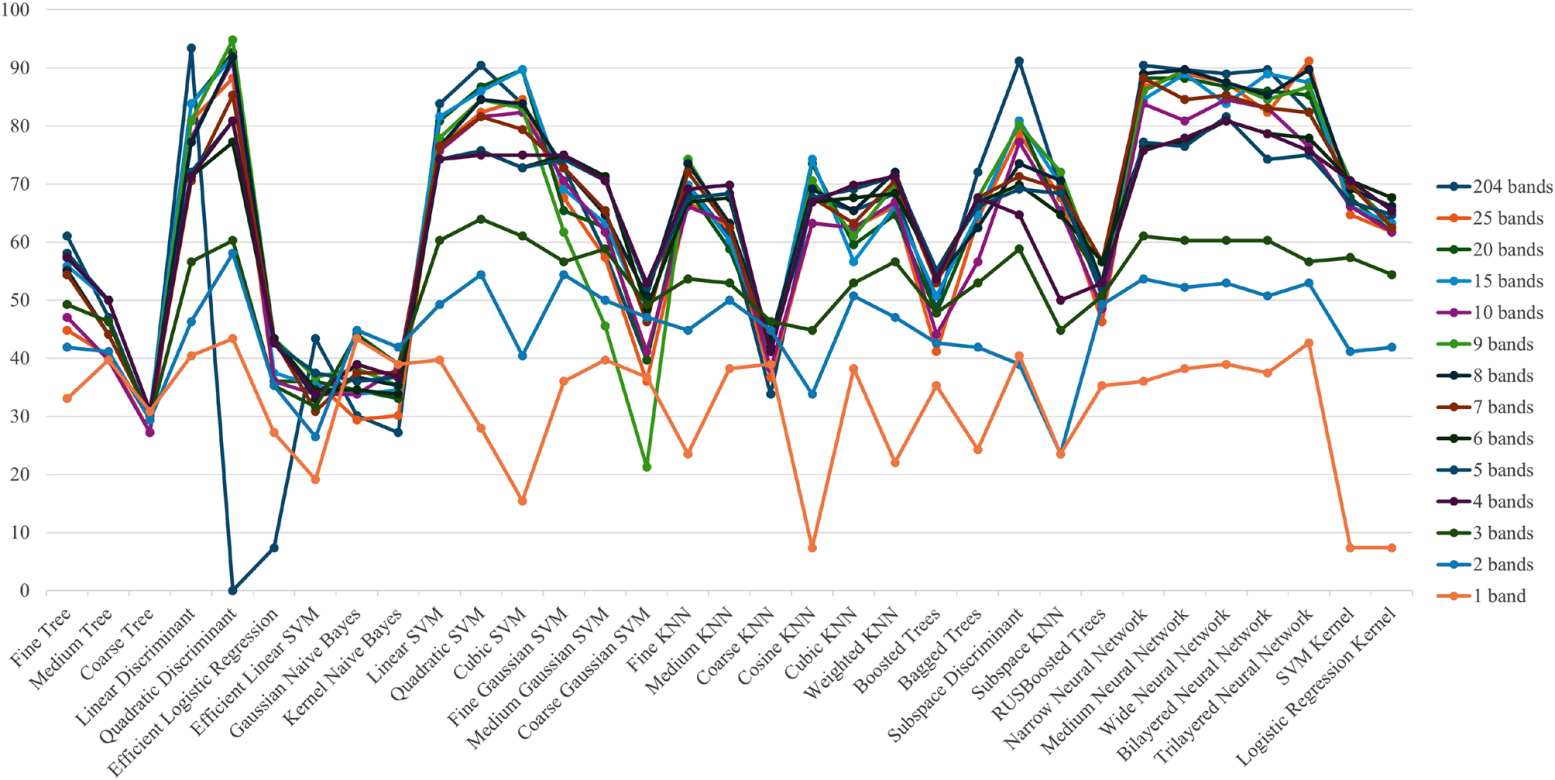
Machine learning results (Test accuracy).

In addition, RGB images (3 bands) and HS data (204 bands) were analyzed in the same manner and compared to the identification accuracy of the MS data. The result is shown in Figs. 8 and 9. The plots include the change in the number of bands selected by the NCA and the corresponding change in the accuracy of mineral determination by machine learning. It is noteworthy that the machine learning models are sequentially selected and presented for the best-performing models as outlined in Table 1. Figures 8 and 9 also compares the discrimination accuracy of the RGB images, HS data, and MS data.

Figure 9 shows that the machine learning identification results for the 15–25 bands of data selected by NCA are high, exceeding 90%. It also shows that the identification accuracy in those bands is almost the same as that of the HS data, indicating that mineral assemblages can be identified with high accuracy with approximately one-tenth or less of the data volume. This suggests the potential to reduce both computational and economic costs. The data analysis of 6 to 10 bands showed a high identification result of more than 80%, while the identification accuracy tended to decrease as the number of selected bands decreased. The analysis of 3-wavelength selection in particular shows lower identification accuracy than that for RGB images that also have 3 bands.

These results indicate that the arsenic minerals used in this study can be identified with more than 80% accuracy by selecting 6 or more bands in the NCA, and, by using 15 or more bands, the identification accuracy is almost equivalent to the analysis of HS data. On the other hand, when the number of bands selected by NCA is less than 5, the accuracy of arsenic concentration identification is significantly reduced, and perhaps a different algorithm (other than the machine learning algorithm employed in this study) may improve the accuracy of identification when using less than 5 bands.

While the identification of specific wavelengths for new ores necessitates the use of a hyperspectral camera, it is important to note that this camera is primarily employed for the training of AI models, rather than for ore sorting. This implies that with the use of a single hyperspectral camera, AI models can be trained for multiple ores, thereby offering a cost-effective solution in the long term.

### Comparison of wavelength selection methods

In this study, NCA was employed as the wavelength selection method. However, as mentioned above, there are a wide variety of wavelength selection methods for HS data. Therefore, a performance comparison of representative wavelength selection methods is conducted here for simplicity. In this article, the following five-wavelength selection methods (MRMR(Minimum Redundancy Maximum Relevance)^59^, Chi-square distribution test^60^, ANOVA^61^, Kruskal–Wallis (KW) test^62^, and ReliefF^63^) were used for comparison, and the accuracy was compared by using a machine learning to identify arsenic concentration only in the selected bands.

To give an overview of each wavelength selection method, MRMR minimizes redundancy by selecting data sets that are not similar for paired data. The Chi-square variance test compares the difference between the variance values of the data and those of the assumed data. ANOVA also analyzes variance but compares significant differences between groups; the KW test is a nonparametric method of ANOVA. RelieF then matches and ranks features between pairs of data.

Each is a different wavelength selection algorithm, and their effectiveness for the data set used in this study compared to NCA is compared. The following Table 3 shows the selected bands ranking by the aforementioned five methods and the NCA.

**Table 3:** Wavelength regions ranked by wavelength selection methods.

Ranking	NCA [nm]	MRMR [nm]	Chi2 [nm]	ReliefF [nm]	ANOVA [nm]	KW [nm]
1	400	403	400	400	997	400
2	994	802	403	994	994	403
3	822	994	406	997	406	406
4	459	400	409	406	403	994
5	828	633	994	403	400	997
6	846	962	997	409	409	409
7	657	415	991	991	991	412
8	580	406	979	985	985	985
9	660	941	985	979	412	979
10	704	459	988	412	979	991

Table 3 shows that among the wavelength bands at 400 ~ 1000 nm, the bands at the ends near 400 nm and 1000 nm tend to be selected mainly by Chi2, ReliefF, ANOVA, and KW methods. While the wavelength bands at the edges of those bands are selected for NCA and MRMR, the bands selected are widely scattered, and this is especially true for NCA. While the Chi2, ReliefF, ANOVA, and KW methods are algorithms that mainly analyze the dispersion values of the data, NCA/MRMR is an algorithm that uses the nearest neighbor method and redundancy, which may be the result of the differences caused by these algorithms.

**Figure 10:**
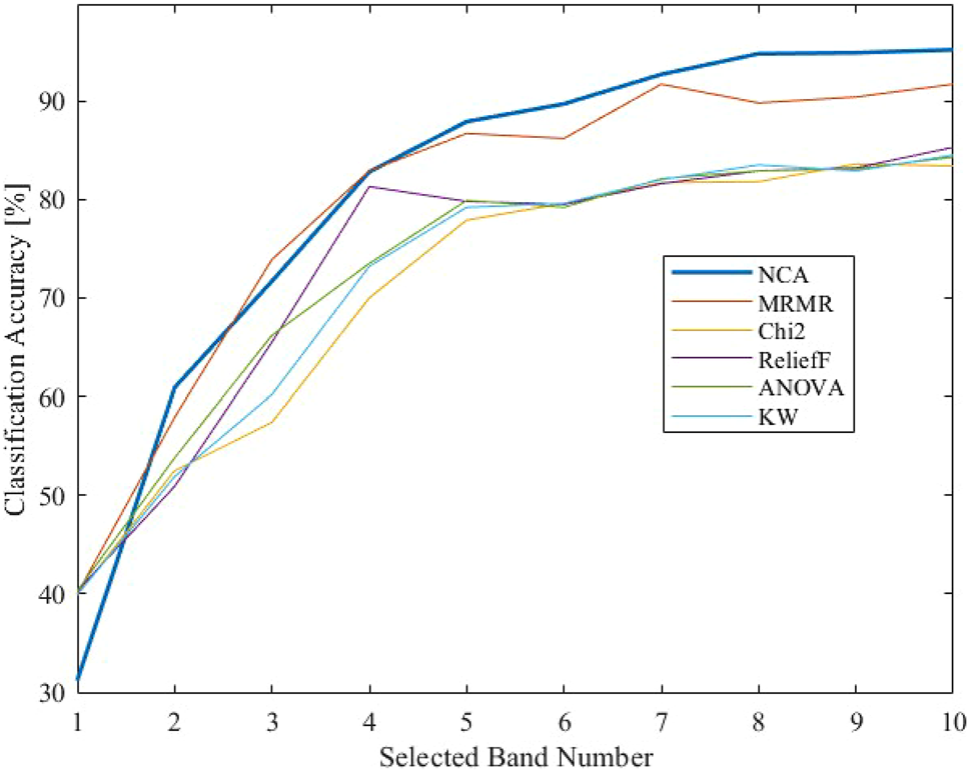
Wavelength selection method comparison of classification accuracy (NCA, MRMR, Chi2, ReliefF, ANOVA, and Kruskal–Wallis).

Since the bands selected by the band selectors were different, they performed machine learning classification using only the wavelength data selected by them and compared which band selector was the best. The number of bands to be trained varied from a ranking of 10 to 1. The learning results are shown in Fig. 10 below.

Figure 10 shows that the NCA method outperformed other wavelength selection methods for the present sample in the band number range of 4–10. Table 3 confirms that this is because other methods react strongly to components at the edge of the acquired wavelengths, such as 400 nm and 1000 nm, which are outside the 400 to 1000 nm region. It is important to note that hyperspectral cameras are less accurate for edge wavelength data due to their functioning. The accuracy of the selection method, other than NCA, may have been affected. This is due to the selection of wavelength bands with large errors and data variations, resulting in lower accuracy. In contrast, NCA selects bands in the wavelength range from visible light to near-infrared light, resulting in higher classification accuracy.

One possible reason is the influence of the absorption spectra in the chosen bands. The absorption spectrum of a mineral can be attributed to two processes, electronic and vibrational^64^, but especially in the short wavelength band, it is caused by the energy order of electron transitions. In the near-infrared wavelength band, absorption spectra are caused by molecular contraction and other processes. In the ore type used in this study, these absorption spectra are thought to be the cause of the characteristic spectral shape.

**Figure 11:**
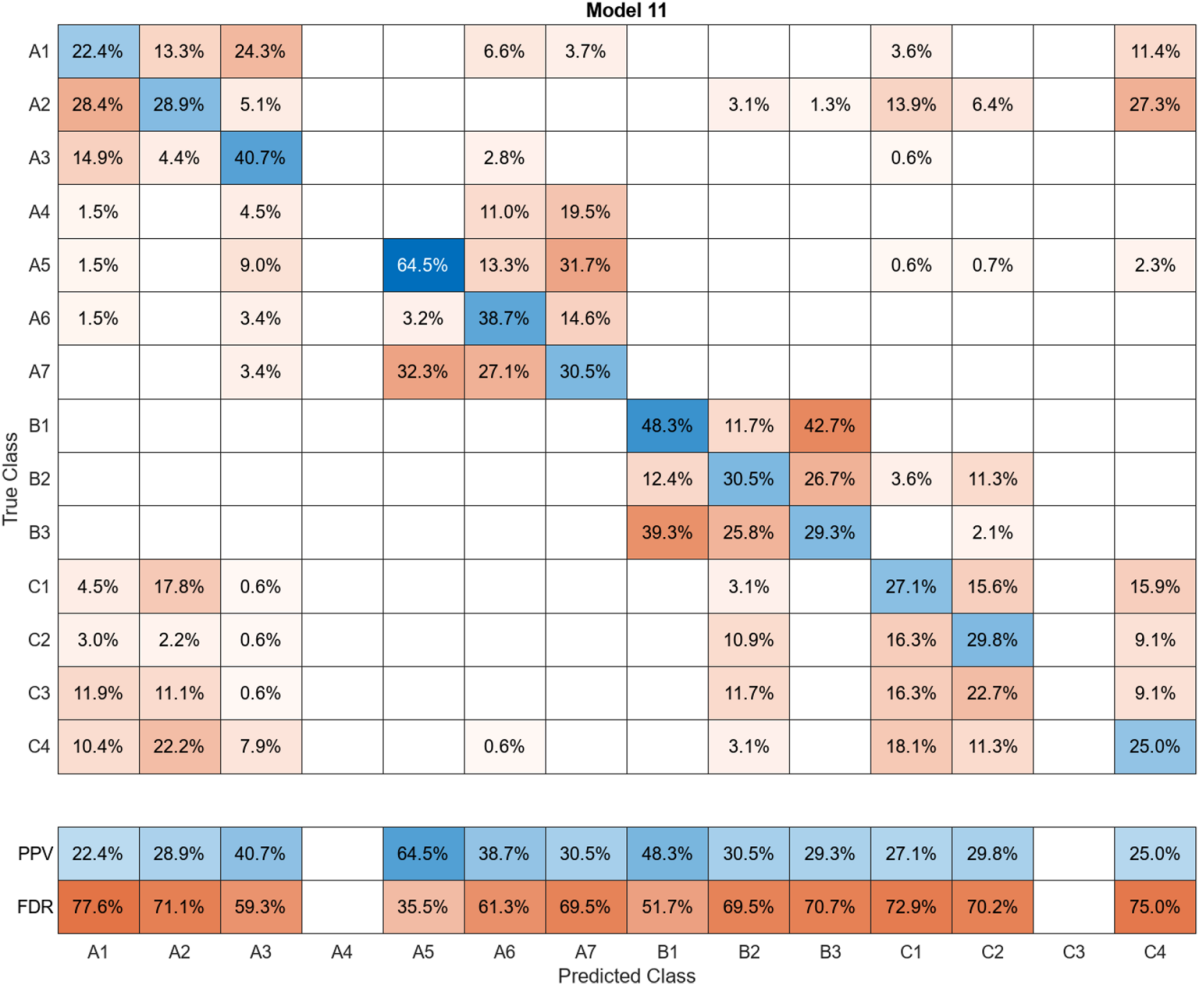
Confusion Matrix of NCA results using 1 band.

**Figure 12:**
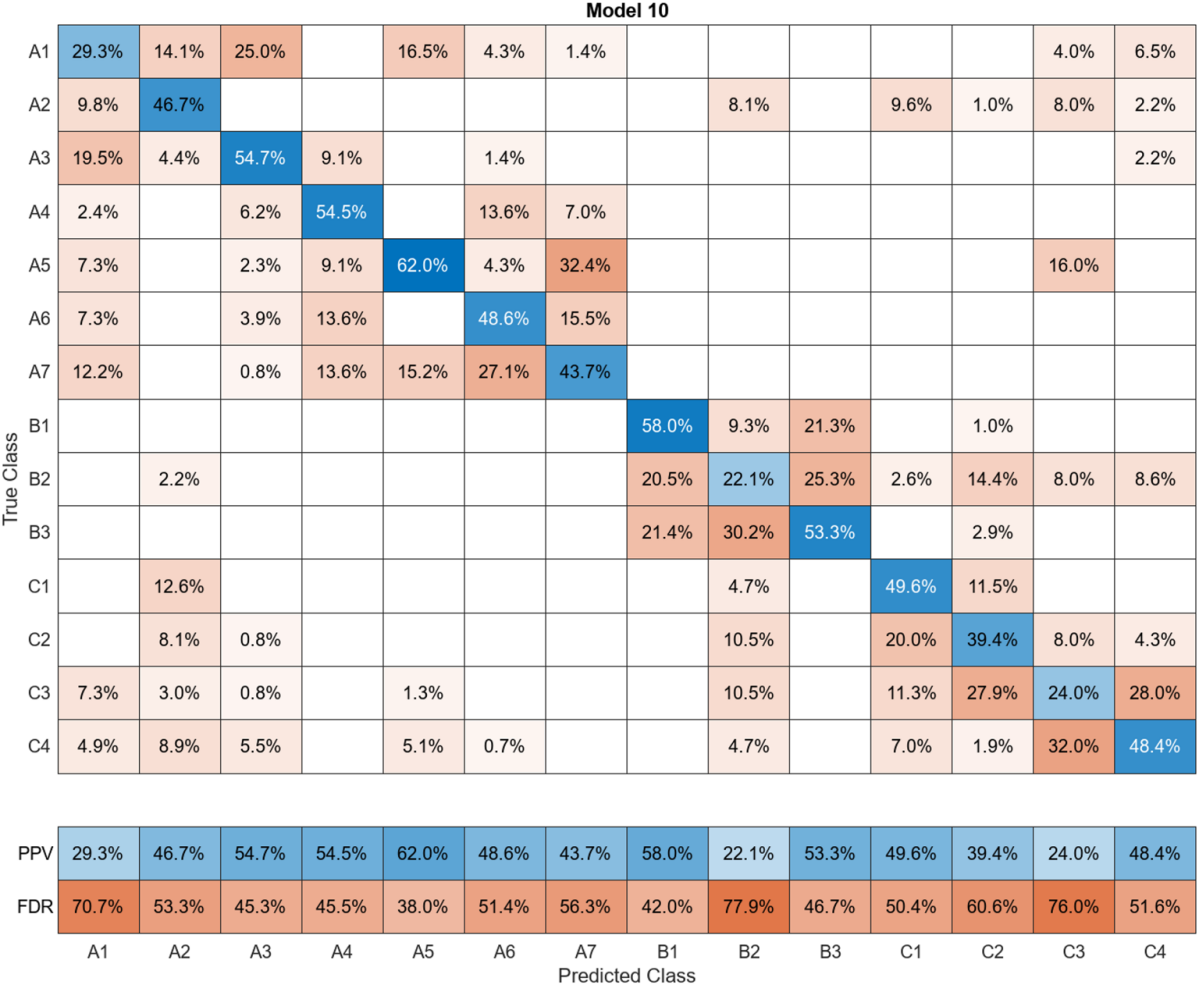
Confusion Matrix of NCA results using 2 bands.

**Figure 13:**
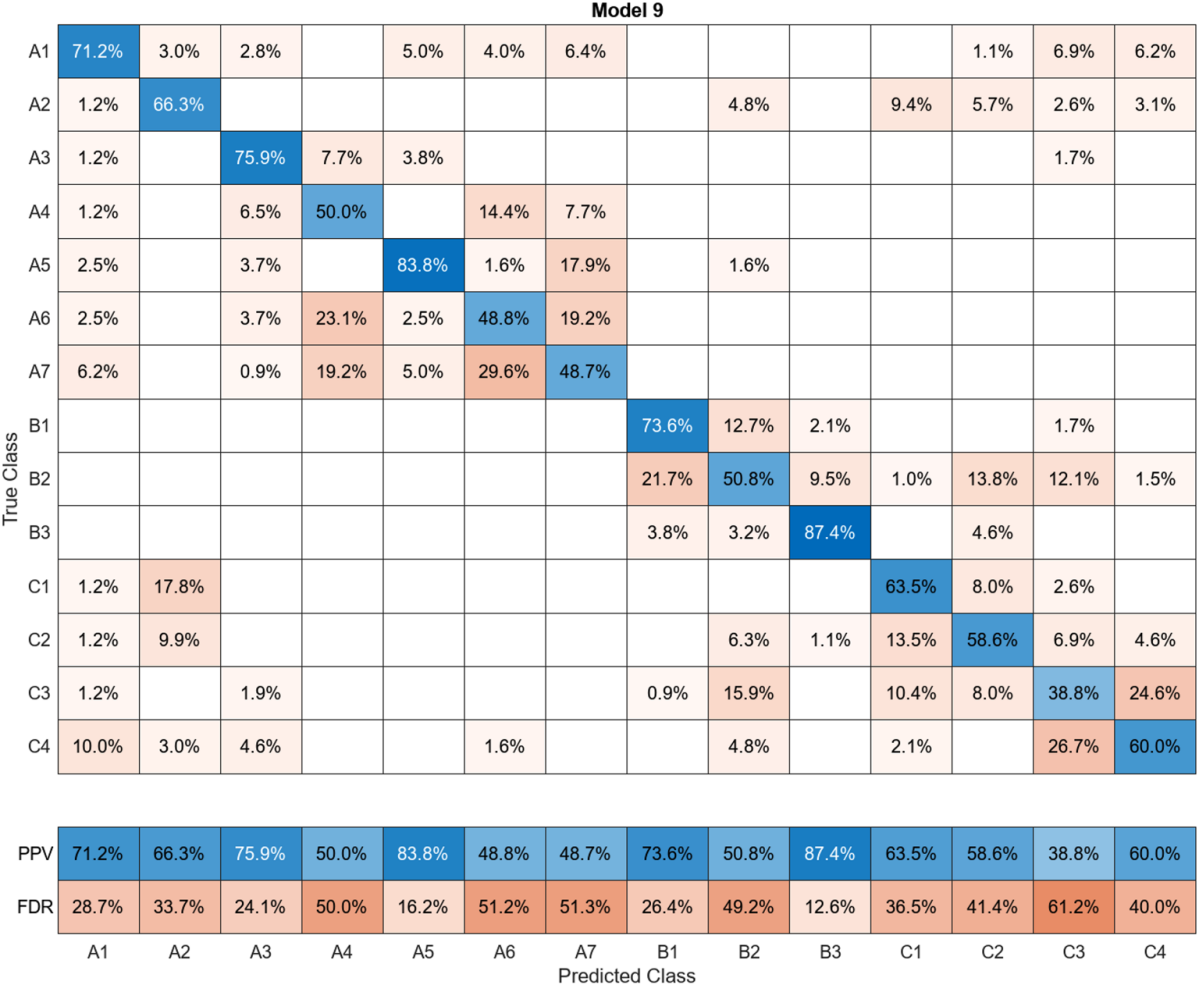
Confusion Matrix of NCA results using 3 bands.

**Figure 14:**
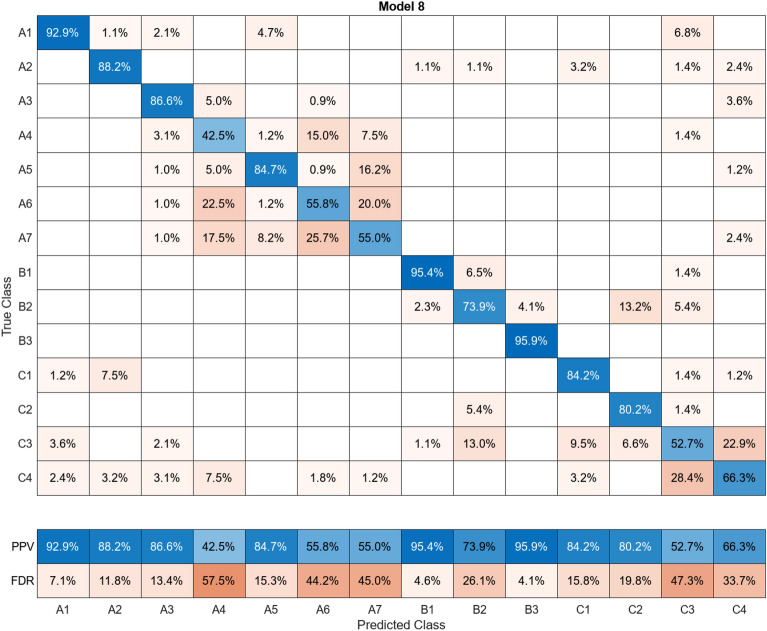
Confusion Matrix of NCA results using 4 bands.

**Figure 15:**
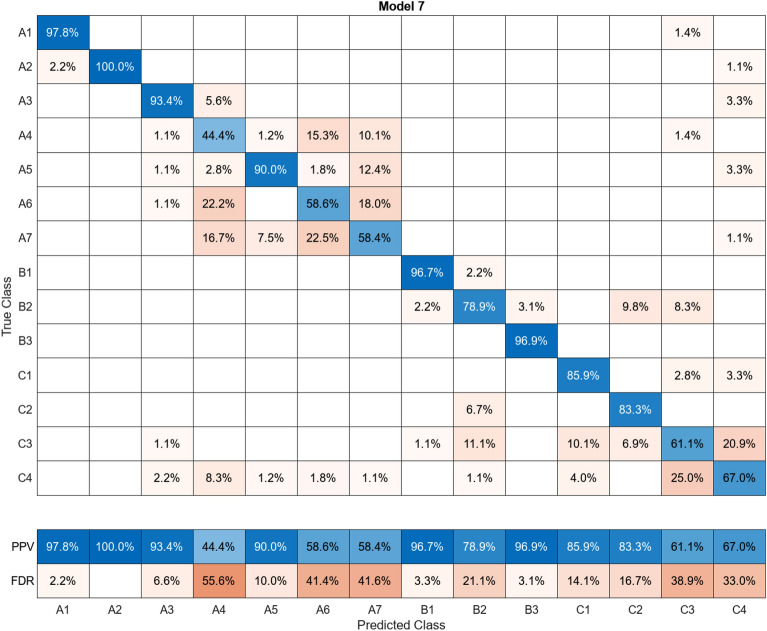
Confusion Matrix of NCA results using 5 bands.

**Figure 16:**
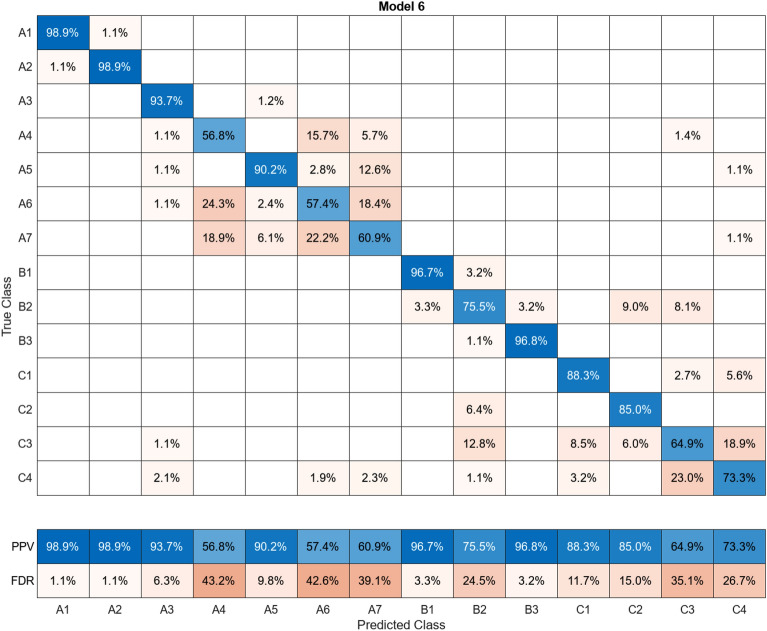
Confusion Matrix of NCA results using 6 bands.

**Figure 17:**
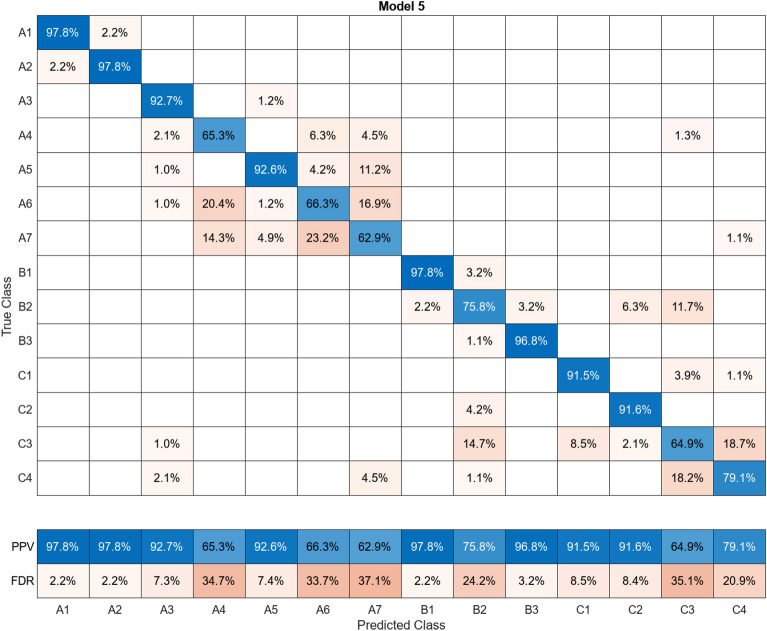
Confusion Matrix of NCA results using 7 bands.

**Figure 18:**
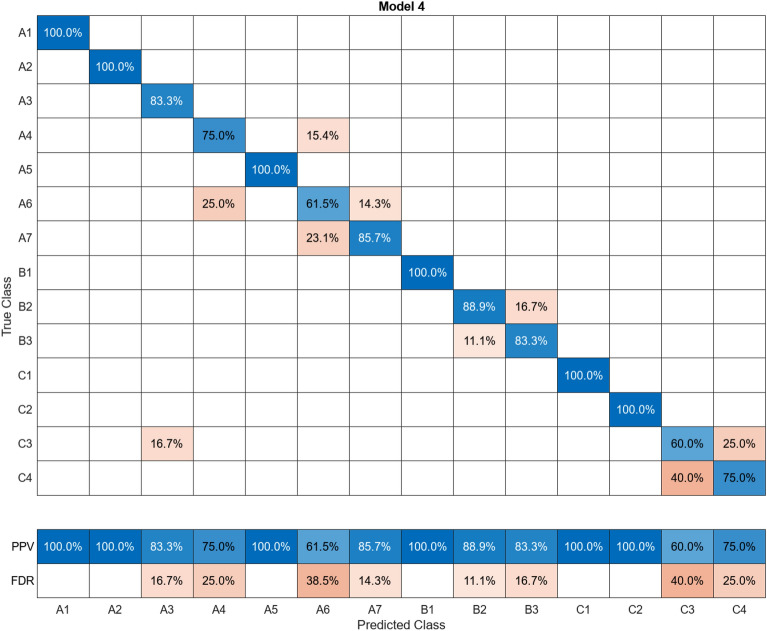
Confusion Matrix of NCA results using 8 bands.

**Figure 19:**
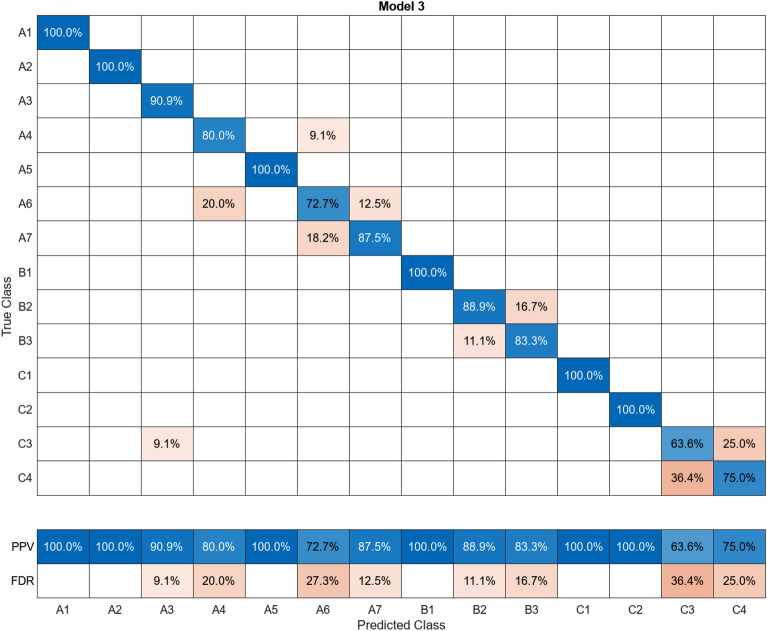
Confusion Matrix of NCA results using 9 bands.

**Figure 20:**
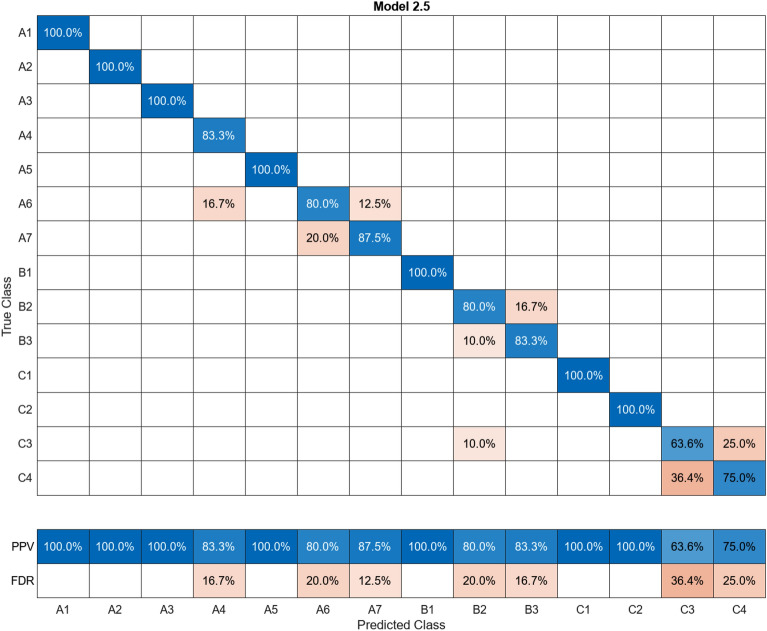
Confusion Matrix of NCA results using 10 bands.

In this study, more than 30 machine learning models were created and tested 15 times each using these models. Despite the limitations of the paper preventing the presentation of the mixing matrices for all of them, Figures (Figs. 11, 12, 13, 14, 15, 16, 17, 18, 19 and 20) show the mixing matrices for the training models trained using only the data reduced in dimensionality using NCA. As illustrated in, the positive predictive values (PPV) values displayed in blue exhibit a concurrent decline with each reduction in the number of bands, whereas the false discovery rates (FDR) values displayed in red demonstrate an upward trend. Additionally, it can be observed that the false discovery rates within the same group tend to increase as the number of bands decreases, whereas the false discovery rates across groups exhibit a comparatively lower increase.

However, the band selector that selected those edge bands cannot be regarded as an excellent band selector because the identification accuracy itself is low (Supplemental materials). Therefore, the wavelength selection method using NCA, which showed an identification accuracy of more than 90% with less than one-tenth of the HS data, was found to be superior by comparison. Furthermore, given that the bands selected by NCA were widely distributed, it was found that there existed not only absorption spectra resulting from vibrational processes of molecules in the far-infrared region in conventional mineral identification, but also characteristic optical property values of the minerals themselves in the visible and near-infrared regions.

Regarding the calculation and processing of HS data, a large amount of calculation resources was required because the data consisted of 204 dimensional wavelengths. In addition, the HS camera itself is expensive, which has been a barrier to its introduction into actual operation. This study shows that identification can be performed by using MS data, which greatly reduces the dimension of data. The Specim IQ HS camera used in this study acquires approximately 300 MB of data in a single shot, whereas MS data acquires only about 3 MB of data. Also, the practical scanning time for Specim IQ is approximately 60 s (depending on the exposure time), while MS cameras can capture images in a few seconds, depending on the type of equipment used. In terms of price, HS cameras are generally expensive, costing tens of thousands of dollars or more, whereas MS cameras are expected to significantly reduce costs. As described above, the dimensionality reduction of HS data using NCA is expected to reduce the amount of data (reduction of computation cost), scan time, and price.

## Limitations and future works

This experiment was conducted in an idealized environment with homogenized and perfect lighting; to improve the classification accuracy by AI and use it in actual operations, the training data must be tuned to improve the robustness of the data. The research may encounter difficulties in extrapolating its findings due to its focus on arsenic-containing minerals found in samples of copper ore. To improve the usefulness of the methodology in various situations, it would be advantageous to include a wider variety of mineral compositions and types of ore in future research. The practicality of incorporating hyperspectral imaging and machine learning algorithms may also be constrained by issues such as equipment availability and cost. Taking steps to address these limitations will lead to a greater acceptance of the methodology within the mining industry. Moreover, discrepancies in the composition of the ore and the prevailing climatic circumstances may influence the efficacy of the suggested approach. Examining the resilience of the technique under various settings would yield vital insights into its practical usefulness.

In order to ascertain the efficacy and feasibility of the suggested approach in real-life scenarios, further research endeavors could encompass the implementation of field trials or pilot studies inside active mining settings. Additional refinement of machine learning algorithms and approaches has the potential to improve the precision and effectiveness of mineral identification, especially in situations that involve intricate ore compositions. Investigating the potential for combining hyperspectral imaging with new sensor technologies may result in more efficient and economical methods for mineral processing and sorting. The environmental implications of the proposed approach, including energy consumption and waste production, must also be evaluated in order to guarantee long-term viability. Creating intuitive interfaces and software tools for analyzing and visualizing data could help mining sector personnel embrace hyperspectral imaging and machine learning approaches.

## Conclusion

The combination of hyperspectral imaging (HS) and sensor-based ore sorting (SBOS) systems, particularly in addressing challenges within the copper extraction industry, presents a promising solution amidst increasing demand for minerals in renewable energy and electric vehicles. The depletion of high-grade ore and the emergence of high-arsenic copper resources pose significant obstacles to sustainable mining practices. Arsenic contamination complicates mineral processing and raises environmental and health concerns. Nevertheless, by incorporating contemporary technologies like hyperspectral imaging into SBOS systems, it becomes feasible to address these difficulties with efficiency. Sensor-based ore sorting systems offer advantages in selectively extracting valuable minerals based on their unique properties, optimizing resource utilization, and reducing processing costs. Hyperspectral imaging enhances these systems by providing detailed mineral characterization, enabling precise mineral identification and classification based on spectral fingerprints. By leveraging techniques like Neighborhood Component Analysis (NCA) for wavelength selection, ore sorting systems can focus on specific spectral bands that are most effective in distinguishing desired minerals, thereby improving efficiency and selectivity.

Machine learning was performed on each of the top 25, 20, 15, and 10 ~ 1 band data sets selected as a result of the NCA analysis to show the change in identification accuracy with the number of bands. By selecting the most accurate data from the multiple machine learning results, statistical errors caused by differences in compatibility between the data and machine learning were eliminated. As a result, the machine learning results for the 6–25 bands selected by NCA were almost the same as the identification accuracy of the HS data with 204 bands. On the other hand, the identification accuracy decreased significantly for bands 1–5. This indicates that arsenic-containing minerals can be identified with a high accuracy of more than 90% by selecting 6 or more bands in the NCA and performing machine learning classification. At the same time, similar tests were conducted using major band selectors other than NCA (MRMR, Chi2, ANOVA, KW, and ReliefF) as verification experiments, and NCA showed the highest performance.

This research has shown that with the integration of the NCA, identification accuracy of arsenic-containing minerals can exceed 90% with just a fraction of the original HS data volume. This approach not only rivals the performance of full HS data but also offers greater efficiency and cost-effectiveness. Comparison experiments with other wavelength selection methods reaffirm the superiority of NCA in terms of classification accuracy and efficiency. Moreover, the ability to customize wavelength selection according to ore characteristics further enhances the adaptability and effectiveness of ore sorting systems. This research underscores the transformative potential of hyperspectral imaging and machine learning in revolutionizing mineral processing and sorting technologies. This strategy addresses the difficulties related to high-dimensional data and enhances the accuracy of categorization. Further research endeavors could explore field trials, refine machine learning algorithms, and investigate new sensor technologies to advance the efficacy and applicability of these methodologies in real-world mining operations.

### Supplementary Information

Supplementary Information.

## Data Availability

The data used in the study are available from the corresponding author upon reasonable request.

## Abbreviations

As: Arsenic
H: Height
HS: Hyperspectral
ICP: Inductively coupled plasma
LOO: Leave-one-out
MS: Multispectral
NCA: Neighborhood component analysis
N: Number of bands
SBOS: Sensor-based ore sorting
W: Width
